# Performance of the pBHR1 mobilization protein MobV and its role in stable plasmid expression in *Rhodopseudomonas palustris* CGA009

**DOI:** 10.1128/spectrum.03819-25

**Published:** 2026-07-16

**Authors:** Mark Kathol, Quin Barton, Cheryl Immethun, Rajib Saha

**Affiliations:** 1 Department of Chemical and Biomolecular Engineering, University of Nebraska-Lincoln 14719, Lincoln, Nebraska, USA; Reichman University, Herzeliya, Israel

**Keywords:** synthetic biology, plasmid stability, plasmid copy number, mobilization protein, relaxase

## Abstract

Mobilizable plasmids are typically used in metabolic engineering studies, especially for their small size, to express heterologous proteins in new host organisms to manipulate their metabolism. *Rhodopseudomonas palustris* is a non-model soil bacterium of interest that is well-known for its extensive metabolic versatility, being able to accumulate a wide range of industrially relevant bioproducts, such as polyhydroxybutyrate, *n*-butanol, hydrogen, and other lignin-derived compounds. However, many of these non-model organisms are more genetically recalcitrant, and the rules of genetic stability, or even plasmid stability, can change drastically from organism to organism. This study investigates the effects of pBHR1’s native mobilization protein, MobV, on the retention of pBBR1 origin plasmids in *R. palustris*, and the effects of supercoil regulation on both plasmid stability, as well as plasmid-based gene expression. Mobilization proteins participate in horizontal gene transfer between bacterial species. Through two functional assays, a relaxation and a conjugation assay, we determine that the relaxation mechanism is similar to a previously annotated mobilization protein, MobM, and confirm that *R. palustris* is able to participate in conjugation using its two native type IV secretion systems, respectively. Using flow cytometry, we determine that mutations to various homologous active sites deleteriously impact plasmid expression. Finally, through RT-qPCR, we also determine that the presence of the mobilization protein confers a large positive effect on copy number, where its absence reduces the copy number from 44.27 ± 2.00 copies per cell to 22.86 ± 0.63.

**IMPORTANCE:**

Plasmid instability remains a major barrier to genetic engineering in non-model gram-negative bacteria, such as *Rhodopseudomonas palustris*. During previous efforts to optimize plasmid vectors for this species, we observed rapid post-transformation unstable plasmid-based expression when using a minimized pBHR1 backbone lacking the native mobilization protein MobV. Restoring MobV eliminated this instability, suggesting an uncharacterized role in plasmid maintenance. In this study, we systematically dissect the contribution of MobV to plasmid expression in *R. palustris* by performing site-directed mutagenesis for several histidine residues. By comparing MobV to the well-characterized relaxase MobM, and generating active-site mutants, we link specific catalytic residues to plasmid persistence, stable genetic expression, and MobV activity. These findings clarify a previously overlooked mechanism of plasmid maintenance in *R. palustris* and provide design principles for constructing stable, high-performing vectors in non-model gram-negative hosts. This work therefore supports more reliable metabolic engineering strategies in organisms of growing biotechnological interest.

## INTRODUCTION

Mobilization proteins participate in horizontal gene transfer, also known as conjugation (1–6). Conjugation is a biological process that necessitates the coordinated action of multiple distinct protein complexes (1). Most bacterial conjugation occurs with the presence of a type 4 secretion system (T4SS), where the bacterium forms a pilus using several of the proteins encoded by the “transfer” or “*tra*” (sometimes “*trb*”) operon. The pilus acts as a channel for the donor bacterium to pass transfer DNA, or other mobilizable or conjugal elements. There are also several differing T4SS architectures (2–4). The specific role of the mobilization protein is to act as a relaxase, performing a single-stranded, phosphodiester bond break, or “nick,” to the plasmid in order to remove supercoiling from a plasmid (5). Mobilization proteins typically originate from small plasmids (<20 kb). These small plasmids are useful for synthetic biologists for providing a quick method of expressing a heterologous protein in an organism capable of maintaining plasmids (7, 8). Mobilizable plasmids contain a mobilization protein and often rely on the host’s endogenous T4SS for transfer (9), and they are more commonly found in gram-positive bacteria (10). A variety of non-model gram-negative bacteria are being investigated for their unique metabolic behaviors; however, many challenges remain as far as ensuring genetic tractability. Because these plasmids are typically smaller to allow for higher transformation efficiency, many engineered, stably replicating plasmids that are employed in metabolic engineering studies are derived from mobilizable plasmids (10–16).

*Rhodopseudomonas palustris* CGA009 (hereafter *R. palustris*) is a gram-negative, non-model bacterium of particular interest to this study. It is known for its metabolic versatility, as well as a high level of enzymatic redundancy. Of relevance to this study, it possesses two different *virB/D4* operons encoding for two T4SSs (*rpa2224–rpa2233* and *rpa4115–rpa4124*) (17). Because of its unique metabolism, there have been many studies which have pursued engineering various *R. palustris* strains for bioproduction of hydrogen, *n*-butanol, polyhydroxybutyrate, and funneling lignin catabolism (17–27). Since the central focus of many metabolic engineering studies is to manipulate the genetic content of an organism to favor human interests, much weight is given to discovering reliable methods and tools to incorporate heterologous DNA. The use of non-endogenous plasmids is widespread in studies involving bacteria, as bacteria are typically more genetically competent and are able to maintain plasmids independently of the chromosome (16, 28). Many of the current genetic manipulation tools, however, are not as effective for non-model organisms such as *R. palustris* (29, 30). As many of these non-endogenous plasmids contain mobilization proteins, there have been some efforts to determine if they are necessary for plasmid maintenance in monocultural conditions (7).

The most common strategy among gram-negative bacteria for the maintenance of non-endogenous plasmids is the use of a high copy number plasmid, whereby there exists such a high number of plasmids within a given cell that it is statistically improbable to produce a daughter cell via reproduction through fission (31–33). A previous study outlined plasmid construction for reliable plasmid expression of heterologous proteins in *R. palustris* (34), utilizing a pBBR1 origin without its endogenous mobilization gene, *mobV*, belonging to the MobV mobilization protein family. However, the resulting plasmid in this study, pSSBIO32, demonstrated a relatively low copy number of plasmids per cell. Plasmid maintenance is also crucial for any plasmid expression of new genetic circuits into the host organism. Because mobilization proteins participate in plasmid topology regulation, it is possible that their activity also influences plasmid copy number, or even regulates plasmid-based gene expression. MobV, originating from the pBHR1 plasmid derived from pBBR1, has high structure and sequence homology with MobM, a previously annotated mobilization protein from the streptococcal plasmid pMV158 (12), which is noteworthy for possessing histidine active sites as opposed to the traditional tyrosines used in most relaxase mechanisms (35).

**Fig 1:**
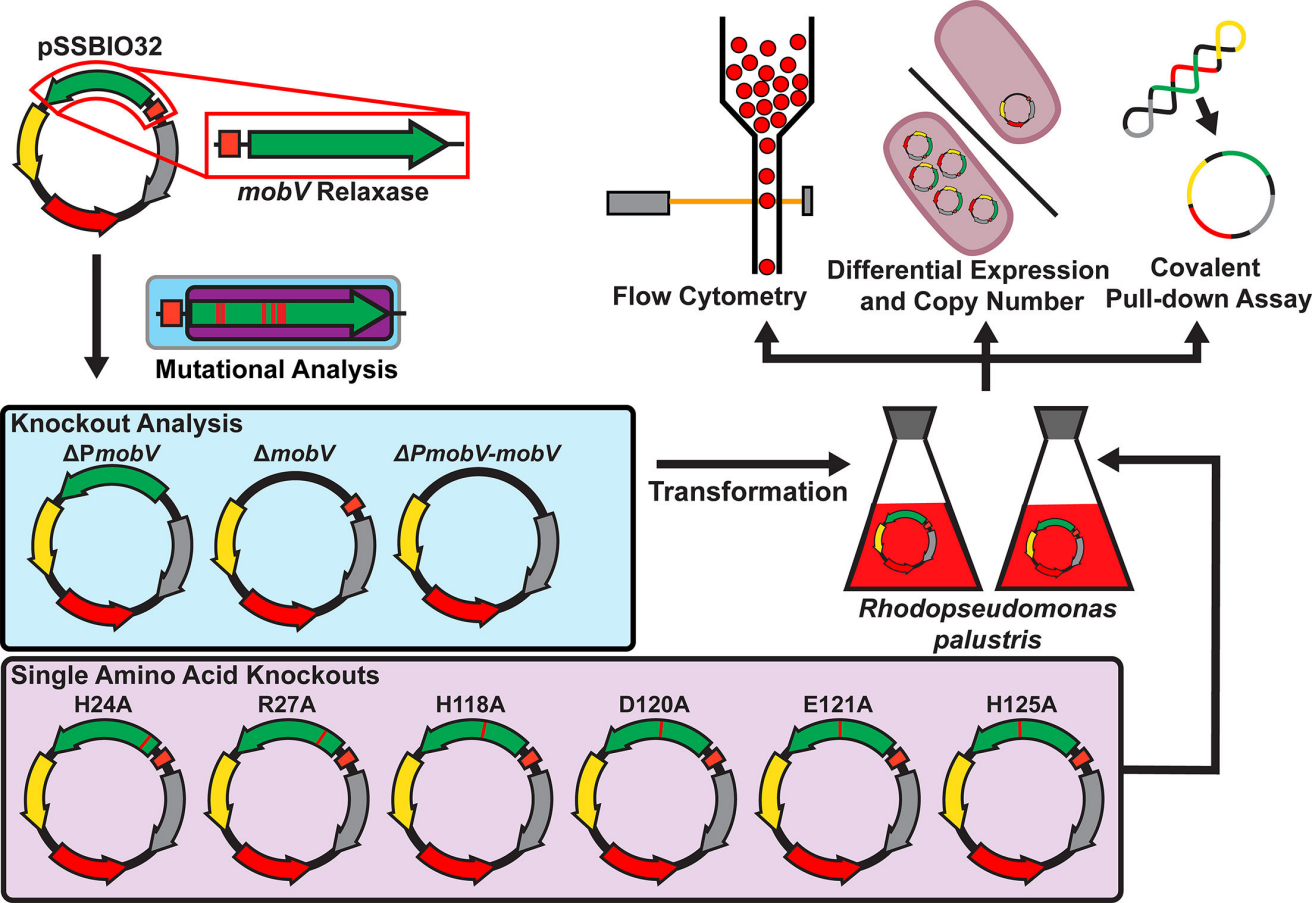
Methodology of testing MobV mutant’s performance in *Rhodopseudomonas palustris*. Vectors containing various mutations to *mobV* were constructed and inserted into *R. palustris*. Cultures were then evaluated through various metrics to determine each mutation’s effect on plasmid stability and gene expression rates. Flow cytometry was used for determining overall plasmid retention rates by cultures, RT-qPCR was used to determine relative expression levels of the different constructs, and proteins of several of the MobV mutants were purified to perform *in vitro* plasmid relaxation assays.

This study will therefore investigate the role of each of the homologous histidine active sites present in MobV, H24, H118, and H125, as well as several other annotated active sites, R27, D120, and E121, in MobV’s relaxase role (Fig. 1). This study will also determine the effect that MobV has on the stability of pSSBIO32, and whether its activity or presence on the plasmid is relevant to maintaining the plasmid through multiple generations. Beyond defining the catalytic contributions of these residues, this work frames MobV as a modular component for improving plasmid stability in non-model bacteria.

## RESULTS AND DISCUSSION

### MobV structure and homology with MobM

**Fig 2:**
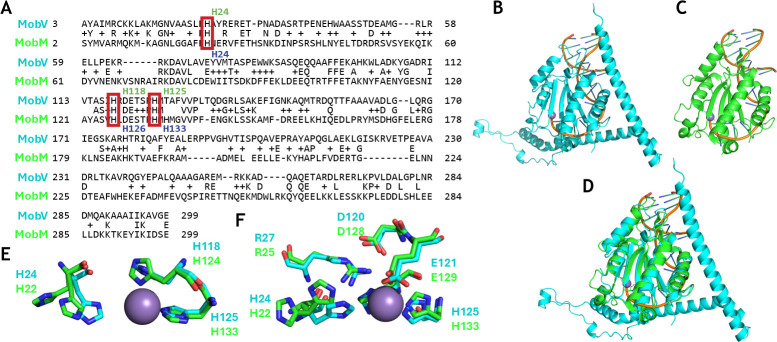
(**A**) Sequence alignment between MobM and MobV. Red boxes highlight histidine residues essential for MobM’s nicking activity and corresponding homologous active sites for MobV. (**B**) AlphaFold (38, 39) predicted crystal structure of MobV obtained from UniProt (40). (**C**) Experimentally determined crystal structure of MobM from *Streptococcus agalactiae* (5). (**D**) Structure alignment between the experimentally determined MobM crystal at pH 6.8 and MobV prediction. (**E**) Aligned active site structures with coordinated Mn^2+^ ion for nicking activity of both proteins exclusively for histidines and (**F**) other active sites essential to MobV.

A relaxase is a topoisomerase enzyme responsible for removing supercoiling from a plasmid before conjugation. Relaxases participate in horizontal gene transfer by assembling with coupling proteins and proteins associated with the bacteria’s T4SS, which allows it to deliver plasmid DNA to the T4SS, to then be propagated to another cell. Relaxases perform the relaxation of plasmid DNA by generating a hairpin-loop structure at a “nick” site to allow the plasmid DNA phosphodiester bond to be cleaved on a single strand, to then allow supercoiling to be removed (36). What is unique about MobM, and other MobV family relaxases, is that they use histidines as the main amino acid residue responsible for carrying out plasmid DNA nicking, whereas most other relaxases traditionally use tyrosines (35, 37). Given that MobV belongs to the same relaxase family as MobM, MobV may use the same DNA cleavage mechanism as MobM (12). Figure 2 demonstrates sequence similarity between the two mobilization proteins, MobV (blue) and MobM (green). The structure of MobM has been determined through X-ray crystallography by Pluta et al. (5), and MobV’s structure is predicted by AlphaFold (38, 39). Despite the large amount of sequence deviation between MobV and MobM (Fig. 2A), the structures between the two proteins are near identical (Fig. 2B through D). MobV importantly retains homology (H24, H118, and H125) between the hypothesized homologous histidine amino active sites present in MobM (H22, H126, and H133). The active site structure (Fig. 2E and F) also retains a near-perfect alignment. In terms of sequence dissimilarity, the largest difference is long alpha helices beginning at L188.

**Fig 3:**
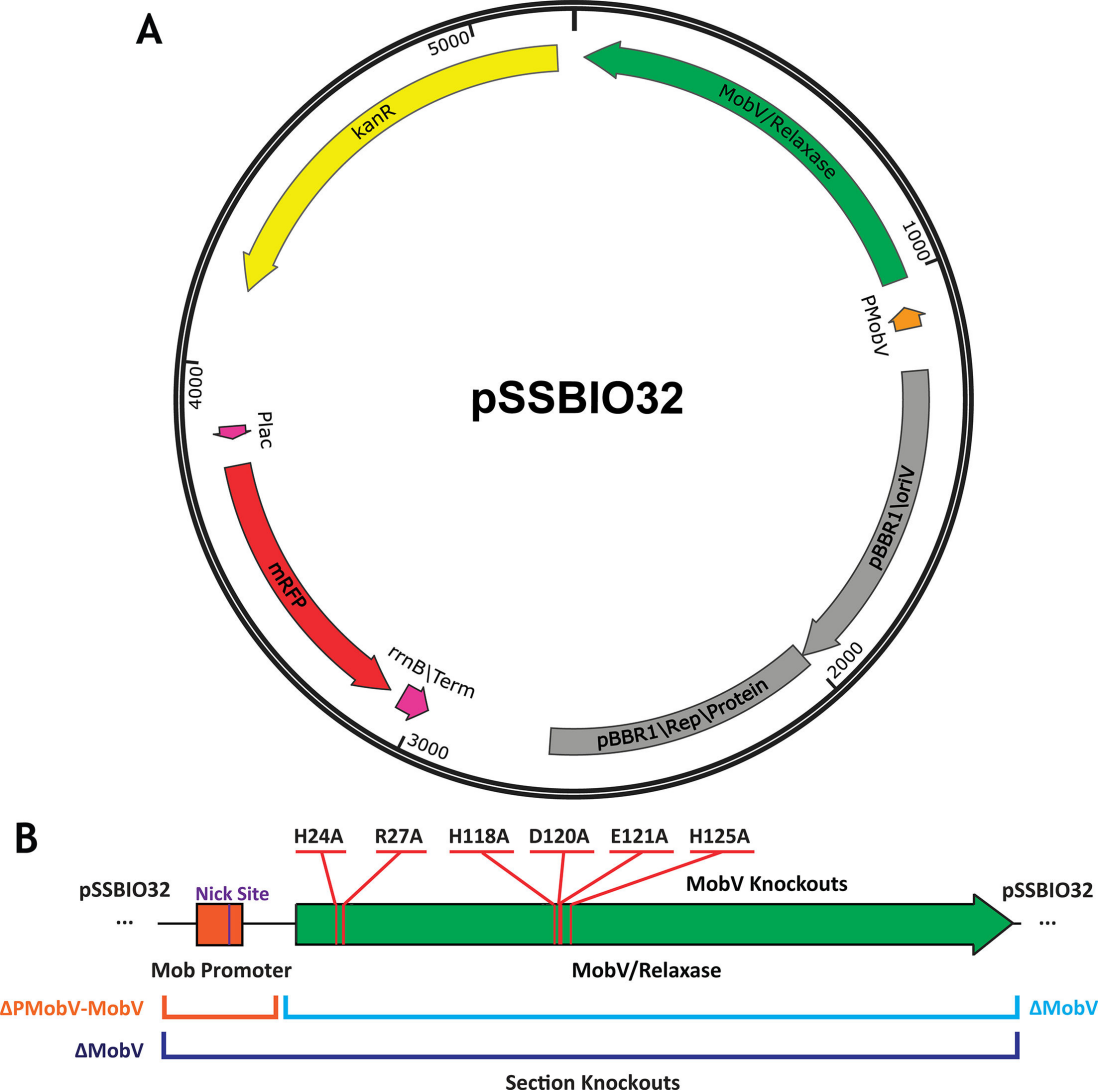
(**A**) Control plasmid used in this study. A pBBR1 origin of replication is used in this study as it has been previously shown to replicate stably in *R. palustris* (34). (**B**) Depiction of site-directed edits of *mobV* and the respective functional knockouts*.*

To test the essentiality of each of the mobilization protein’s active sites and the effects of the knockouts when employed in *R. palustris*, site-directed mutagenesis was performed on MobV (Fig. 3A) to individually remove the activity of each active site (41). To thoroughly test the activity of MobV with each of the active site knockouts, the pSSBIO32 plasmid developed from the previous study was used to maintain its expression in *R. palustris* (34). pSSBIO32 consists of a pBBR1 origin with an mRFP fluorescent reporter, a kanamycin resistance gene for its previously reported efficacy as a selection marker for *R. palustris* at 300 µg/mL, and the MobV coding region (34, 42). Given the amount of similarity between the two relaxases, MobV’s homologous histidines (H24, H118, and H125) were knocked out by replacement with alanine, generating MobV variants H24A, H118A, and H125A. Because the overall structure of the active site is largely conserved, additional residues near the site that were previously annotated as essential for MobV function (R27, D120, and E121) were also knocked out, generating the variants R27A, D120A, and E121A (41). In addition, three deletion mutants were constructed: one lacking the entire mobilization protein-coding region along with its promoter (ΔP*mobV-mobV*), one lacking only the promoter region—which also includes the oriT (ΔP*mobV*)—and one lacking only the MobV coding sequence (Δ*mobV*). These comprised nine total mutants for which subsequent experiments were performed (Fig. 3B).

### Flow cytometry and plasmid retention rates

**Fig 4:**
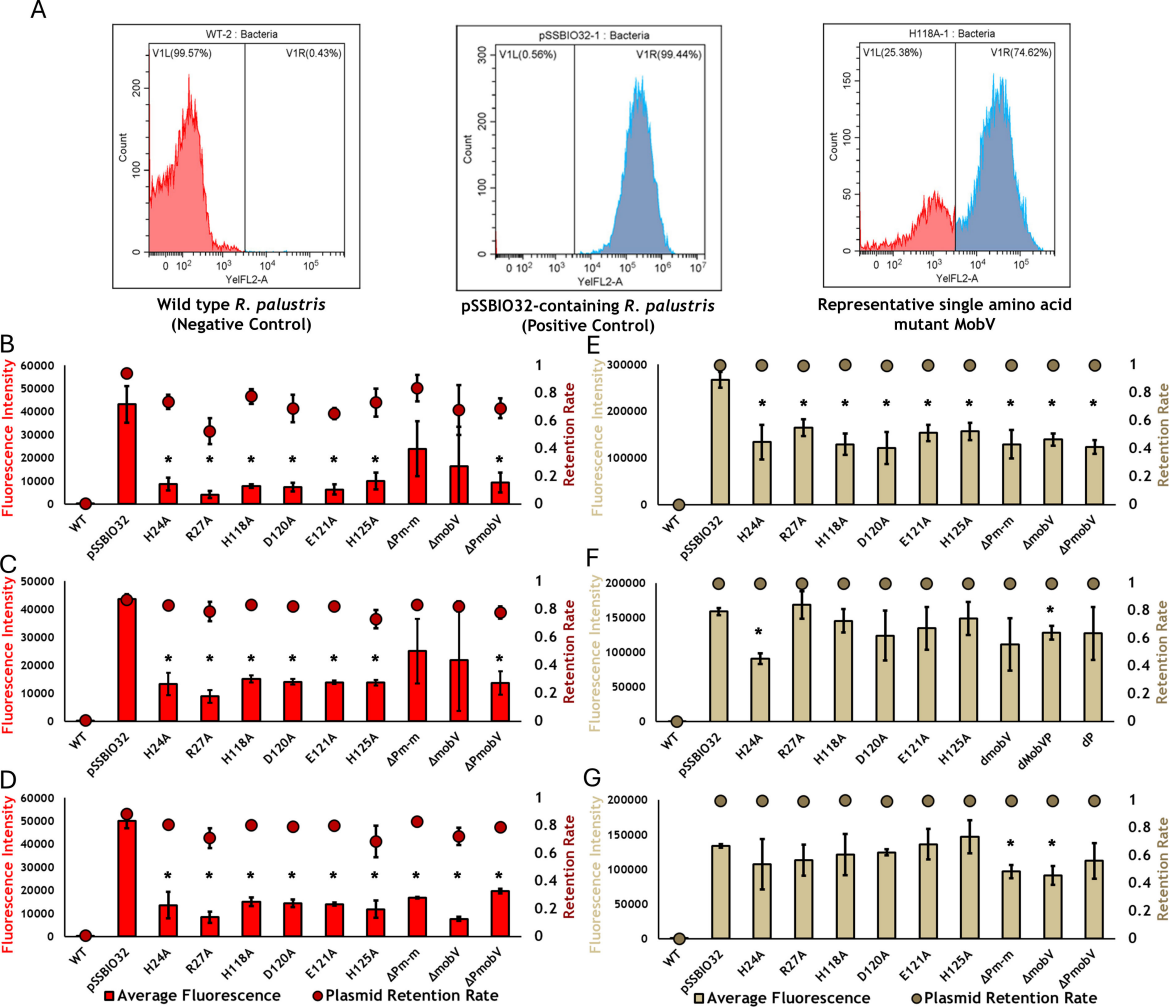
(**A**) Flow cytometry histograms for mRFP fluorescence on each MobV mutant pSSBIO32 plasmid. Left: Example of a wild-type *R. palustris* culture without an *mrfp* fluorescent tag, serving as a negative control. Middle: Example of a transformed *R. palustris* culture with pSSBIO32 containing an unmodified *mobV* gene, serving as a positive control. Right: Example of a transformed culture with a pSSBIO32 plasmid with a mutation to *mobV*. (**B–D**) Flow cytometry results for *R. palustris* cultures after (**B**) 4 days, (**C**) 8 days, and (**D**) 12 days of incubation. (**E–G**) Flow cytometry results for *E. coli* cultures after (**E**) 1 day, (**F**) 2 days, and (**G**) 3 days of incubation. Bars represent the whole culture average fluorescence intensity (left axis), and circles represent corresponding plasmid retention rates (right axis) for *n* = 3. Error bars for both fluorescence and plasmid retention represent one standard deviation. Asterisks represent significant differences in fluorescence intensity between the individual mutants and the positive control vector, pSSBIO32. Plasmid retention rates were consistent to the point where they are not easily visible, and standard deviations can be instead found in Table S1. *P* values for determining significant differences according to Welch’s *t*-test for *n* = 3 can also be found in Table 1.

To determine the effect of each of the individual active site knockouts on retention rates of a bacterium as resistant to antibiotics as *R. palustris*, we transformed both *R. palustris* and *E. coli* with plasmids containing MobV variants. We also transformed *E. coli* DH10β, as it is not as resistant to kanamycin, nor does it possess a canonical T4SS. This was done to simultaneously determine the potentially negative contribution of *R. palustris*’ naturally high antibiotic resistance to plasmid stability, as well as the presence of its two unique T4SSs. Both the average fluorescence intensity of the mRFP fluorescent protein and the overall plasmid retention rates were obtained using flow cytometry (Fig. 4A).

**TABLE 1:** Plasmid retention rates and average fluorescence intensities of *E. coli* and *R. palustris* transformed with pSSBIO32 and pSSBIO32 derivatives containing mutations to MobV^*a*^

*R. palustris*												
	Plasmid retention						Fluorescence intensity					
	Day 4		Day 8		Day 12		Day 4		Day 8		Day 12	
Strain	Average (%)	Significance	Average (%)	Significance	Average (%)	Significance	Average (10^3^ a.u.)	Significance	Average (10^3^ a.u.)	Significance	Average (10^3^ a.u.)	Significance
pSSBIO32	94.4 ± 15	NA	86.7 ± 0.7	NA	88.6 ± 0.5	NA	43.2 ± 7.9	NA	43.7 ± 1.8	NA	50.0 ± 3.1	NA
H24A	73.8 ± 5.1	*	82.6 ± 2.0	–	80.5 ± 2.7	*	8.6 ± 2.7	*	13.3 ± 4.1	**	13.6 ± 5.7	**
R27A	52.6 ± 9.2	*	78.3 ± 6.9	–	71.0 ± 7.3	–	4.1 ± 1.5	*	8.8 ± 2.3	***	8.4 ± 2.4	***
H118A	77.6 ± 5.3	*	82.8 ± 1.5	*	80.3 ± 2.5	*	7.8 ± 0.8	*	15.1 ± 1.2	***	15.1 ± 1.9	***
D120A	69.0 ± 9.9	–	81.7 ± 0.9	*	79.4 ± 1.5	*	7.3 ± 1.8	*	14.1 ± 1.0	***	14.3 ± 1.7	***
E121A	65.2 ± 4.0	*	81.8 ± 0.8	**	79.8 ± 0.6	***	6.3 ± 2.3	*	13.9 ± 0.7	**	14.1 ± 0.7	**
H125A	73.2 ± 10.1	–	72.7 ± 6.5	–	68.6 ± 11.3	–	10.0 ± 3.7	*	13.8 ± 1.0	***	11.8 ± 3.8	**
ΔP*mobV -mobV*	83.7 ± 9.6	–	82.9 ± 1.1	*	82.9 ± 0.3	***	23.9 ± 11.9	–	25.0 ± 11.5	–	16.9 ± 0.3	**
Δ*mobV*	67.9 ± 18.1	–	82.0 ± 3.7	–	72.3 ± 6.2	–	16.3 ± 17.2	–	21.8 ± 18.3	–	7.6 ± 0.9	**
ΔP*mobV*	69.0 ± 7.2	*	77.6 ± 4.2	–	78.8 ± 1.7	*	9.3 ± 4.4	*	13.6 ± 4.2	**	19.7 ± 1.0	**

*E. coli*												
	Plasmid retention						Fluorescence intensity					
	Day 1		Day 2		Day 3		Day 1		Day 2		Day 3	
Strain	Average (%)	Significance	Average (%)	Significance	Average (%)	Significance	Average (10^3^ a.u.)	Significance	Average (10^3^ a.u.)	Significance	Average (10^3^ a.u.)	Significance
pSSBIO32	99.4 ± 0.2	NA	99.5 ± 0.2	NA	99.3 ± 0.6	NA	267 ± 17	NA	159 ± 5	NA	133 ± 3	NA
H24A	99.3 ± 0.1	–	99.6 ± 0.2	–	99.8 ± 0.2	–	134 ± 37	*	91 ± 8	**	107 ± 36	–
R27A	99.1 ± 0.3	–	99.5 ± 0.1	–	99.8 ± 0.1	–	165 ± 18	**	168 ± 20	–	113 ± 22	–
H118A	99.6 ± 0.3	–	99.6 ± 0.1	–	99.7 ± 0.1	–	129 ± 23	**	145 ± 17	–	121 ± 30	–
D120A	99.1 ± 0.2	–	99.6 ± 0.1	–	99.7 ± 0.2	–	121 ± 34	*	124 ± 36	–	124 ± 4	–
E121A	99.4 ± 0.1	–	99.6 ± 0.1	–	99.7 ± 0.1	–	153 ± 18	**	135 ± 31	–	136 ± 22	–
H125A	99.3 ± 0.1	–	99.5 ± 0.0	–	99.4 ± 0.3	–	157 ± 18	**	149 ± 24	–	147 ± 24	–
ΔP*mobV -mobV*	99.4 ± 0.4	–	99.6 ± 0.3	–	99.3 ± 0.5	–	129 ± 31	*	111 ± 38	–	97 ± 9	*
Δ*mobV*	99.5 ± 0.2	–	99.4 ± 0.1	–	98.0 ± 2.6	–	139 ± 13	**	128 ± 10	*	91 ± 13	*
ΔP*mobV*	99.3 ± 0.3	–	99.5 ± 0.1	–	99.2 ± 0.8	–	123 ± 15	**	127 ± 38	–	112 ± 26	–

^*a*^ Significance represents differences between mutant strains and the pSSBIO32 positive control. * = *P* < 0.05, ** = *P* < 0.005, and *** = *P* < 0.0005 for *n* = 3. –, no significance between strain and positive control of pSSBIO32. NA, significance test not applicable. Data can also be found in Data S1 and S2.

Flow cytometry was then performed on transformant cultures after incubation at 30°C and 275 rpm in VN media for 4, 8, and 12 days for *R. palustris*, and LB media for 1, 2, and 3 days for *E. coli. R. palustris* was allowed a longer incubation period than *E. coli* to reach a stationary growth phase due to its much longer doubling time (11 h vs 20 min). The behavior of the different transformants was measured in two metrics, the first being the average fluorescence of the mRFP protein from the entire transformant culture. The second metric measuring performance of the plasmid in the culture was the overall retention rate, which was determined by setting an arbitrary fluorescence threshold (determined to be at ~3,500 a.u.), with cells having fluorescence intensity over this value being considered to retain the plasmid. The overall proportion of each culture retaining its respective plasmid was then determined by obtaining the proportion of the total culture that exceeded, or was to the right of the threshold, on each of the collected histograms.

Figure 4 shows the results of each day where flow cytometry was performed. Transformed *R. palustris* and *E. coli* cultures had markedly different behavior from each other based on these two metrics. *R. palustris* containing the base pSSBIO32 vector with an unmodified *mobV* gene had stable expression over the incubation period in the regime of ~45,000 a.u. (Table 1). However, in *E. coli*, pSSBIO32 provided a much higher fluorescence intensity overall for the first day after transformation (267 ± 17 a.u.), and each of the individual *E. coli* mutants had produced a significantly lower fluorescence intensity. This initial discrepancy between the positive control and the mutants, however, appeared to decrease over time, as the initial fluorescence intensity of the *E. coli* pSSBIO32 culture decreased from day 1 (267 ± 17 a.u.) to day 2 (159 ± 5 a.u.) and remained at relatively the same regime on day 3 (133 ± 3 a.u.). On day 2, each of the mutants in *E. coli,* with the exception of H24A (91 ± 8 a.u.) and *mobV* (128 ± 10 a.u.), had failed to produce a significant difference in fluorescence intensity, while on day 3, only the *ΔPmobV-mobV* (97 ± 9 a.u.) and *mobV* (91 ± 13 a.u.) mutants had produced significantly lower fluorescence. In summary, most of the mutants in *E. coli* had matched the behavior of the positive control strain after 2 days, while in *R. palustris*, the discrepancies persisted. In terms of plasmid maintenance, many of the mutant *R. palustris* cultures had produced significantly lower plasmid retention rates than the pSSBIO32 culture on each day, whereas the *E. coli* mutant cultures produced no significant differences in plasmid retention rates at any time point, with each mutant still providing >98% retention rates.

The difference in fluorescence intensity between the two organisms demonstrates that while the mobilization protein may not improve the performance of vectors in some hosts like *E. coli*, it may be necessary to improve the performance of vectors in other less-tractable organisms such as *R. palustris*. Given that the presence and functionality of MobV produced markedly different effects on plasmid expression in two different gram-negative hosts, MobV appears to exert a substantial host-dependent regulatory influence on plasmid-derived gene expression. MobV primarily functions in plasmid relaxation during conjugation and therefore plays an important role in regulating plasmid topology, which can in turn influence transcription and translation of the plasmid mRNAs, as well as replication of plasmid DNA. Several mechanisms could explain why MobV appears necessary for plasmid expression performance in *R. palustris* as opposed to *E. coli*. One possibility is that MobV directly influences plasmid copy number in *R. palustris* but not in *E. coli*. Alternatively, plasmid copy number may already be sufficiently high in *E. coli*, whereas loss or dysfunction of MobV could lead to a detrimental reduction in plasmid copy number in *R. palustris* to the point where plasmid loss is made much more probable because of the lower copy number. A second possibility is that because of MobV’s function as a relaxase that introduces a site-specific single-stranded nick in the plasmid DNA, the absence of functional MobV could leave a higher proportion of plasmids in a supercoiled state, potentially impairing replication or reducing transcriptional accessibility.

### Functional assays

**Fig 5:**
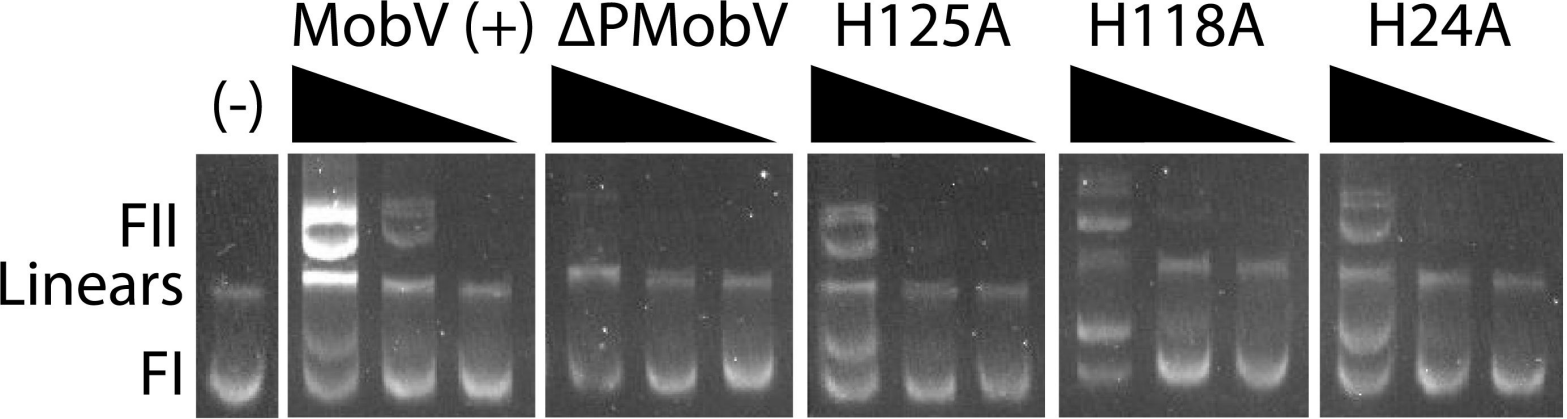
Covalent pull-down assays of histidine MobV mutants. (-) Represents a negative control reaction performed without the addition of MobV protein. (+) Represents a positive control reaction performed with the addition of unmodified MobV protein. Each group of three lanes represents a reaction gradient prepared with 500 ng of supercoiled pSSBIO32 plasmid with 500, 250, and 100 nM of corresponding MobV mutant protein added. FI represents closed-form supercoiled plasmids not relaxed by MobV mutants. FII represents open-form plasmids that were relaxed by each individual MobV protein.

Given that the primary function of mobilization proteins is to perform relaxation, or a single-stranded cleavage of a plasmid sequence, a simple method to determine if the homologous histidine active sites are responsible for cleavage is a pull-down assay. Figure 5 depicts the activity of the individual histidine mobilization protein mutants in this study. A covalent pull-down assay was performed to determine each of the mobilization protein mutants’ ability to “nick” or relax the plasmid by performing cleavage on a single strand of the plasmid at the oriT. In this assay, supercoiled plasmid extract was incubated with MobV proteins and the various developed mutant MobV proteins. Reaction products were then loaded onto an agarose gel to visualize any relaxosomes formed following incubation.

The pull-down assays determine if the specific histidines are crucial to the nicking activity of MobV. If a mobilization protein mutant for a specific amino acid residue fails to relax the plasmid, then that residue is considered crucial to MobV’s activity. Supercoiled plasmids migrate faster during gel electrophoresis than relaxed plasmids; therefore, if the mobilization protein is completely disabled by a deleterious mutation, then the plasmid relaxation assay will ideally produce no relaxed plasmids and will be visually similar to the negative control where no mobilization protein is added. The previous study with MobM demonstrated that removal of specific active sites, most notably the H22 active site present in MobM, resulted in a complete elimination of relaxase activity (41). Should a MobV mutant prove not to be disabled, then it would ideally have the same activity as the unmodified MobV protein (+). From our results, it appears that the removal of any of the critical histidines significantly ablates but does not outright remove the activity of the individual MobV proteins. The removal of strictly the promoter sequence (ΔP*mobV*) appears to act identically to the negative control, which is expected as the promoter sequence also encodes for the oriT, which is the site where MobV performs the relaxation activity. Its removal therefore means that MobV cannot relax the plasmid, and none of the individual mutations do not appear to completely disable the activity of MobV. Even though no open forms can be seen in the 250 nM treated reactions, some open forms can still be seen with the 500 nM treated reactions. This largely appears to agree with the previous study detailing the structure of MobM, which demonstrated that the H22A mutant, the homologous site to H24 in MobV, resulted in a complete elimination of relaxation activity. The largest difference between the generated mutants of this study and of the previous study of MobM is that the highest concentrations of 500 nM were still able to produce nicked plasmids. While there does appear to be a qualitative difference between the positive control of the unmodified MobV protein and the rest of the mutants, a complete ablation was not yet achieved. This could be owing to the fact that the MobV protein does not share a completely identical sequence to MobM, containing only a 28.81% identity, and that nearby amino acids with similar properties, in this case a similar basic amino acid, could compensate for the lost activity of the histidine. This has been precedented in multiple studies involving relaxase activity as well (43–45).

**Fig 6:**
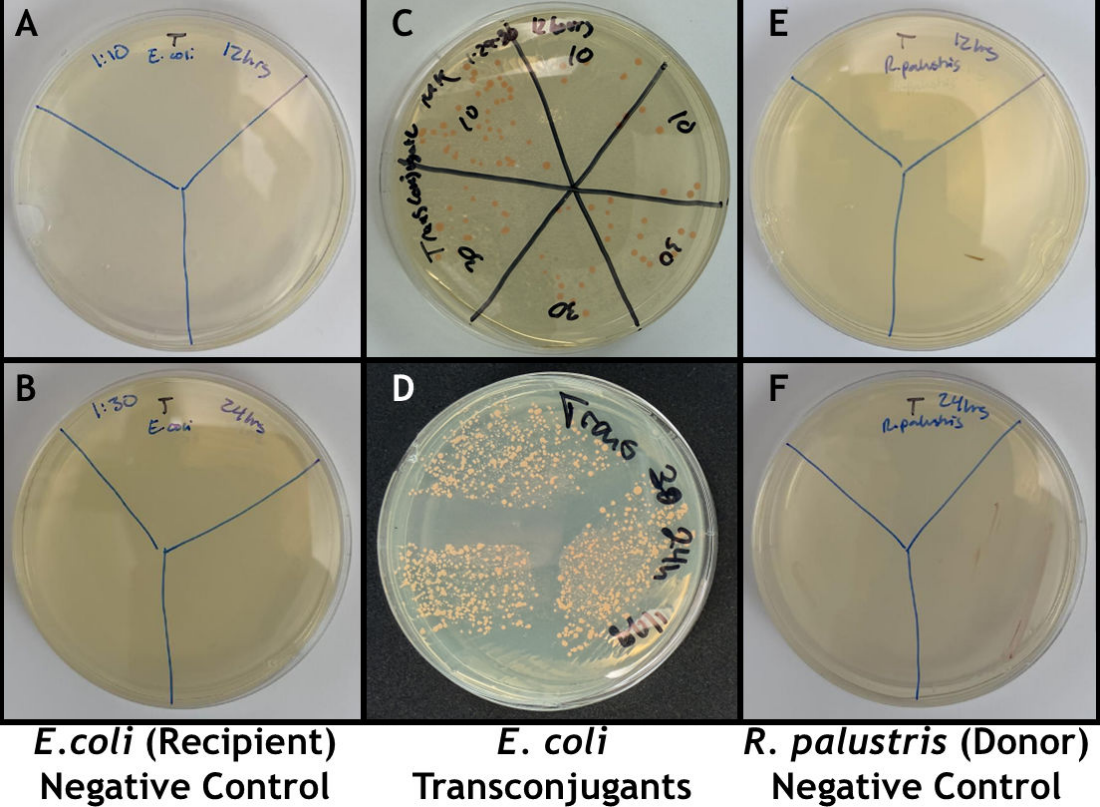
Agar plates after 3 days of incubation at 30°C. (**A**) Negative controls for *E. coli* cultures to ensure selective conditions with a recovery incubation time of 12 h and (**B**) 24 h. (**C**) Transconjugants of *E. coli* grown at 1/30th OD compared to the initial *R. palustris* OD_660_ = 0.2 with (**C**) 12 h recovery time and (**D**) 24 h recovery time. (**E**) Corresponding negative controls for *R. palustris* only cultures with a recovery time of 12 h and (**F**) 24 h.

In addition to the mobilization protein relaxase assay, where we determined if individual mobilization protein mutants are able to still perform the relaxation ability, we also determined if *R. palustris* was in fact participating in conjugation during growth. This experiment was primarily performed to determine if either of *R. palustris* T4SS encoding regions, despite their presence on the genome, are indeed active, as it is possible for organisms to possess conditionally inactive T4SSs (46). For this, we performed a simple conjugation assay protocol with gentamycin-resistant *E. coli*, and *R. palustris* transformed with pSSBIO32 containing an unmodified mobilization protein (Fig. 6). We have therefore prepared transconjugant plates that select only for *E. coli* transformants that have obtained the kanamycin resistance marker from *R. palustris*, which would only be possible through conjugation. Given *R. palustris*’ high antibiotic resistance, the plates were formulated to contain simultaneously kanamycin and gentamycin to constitute stronger selective conditions and were composed of LB media rather than VN, as VN is *R. palustris*’ preferred rich media.

Figure 6A and B demonstrates selection against the gentamycin-resistant *E. coli*, and Figure 6E and F shows the negative controls for the conjugation assay where *R. palustris* transformed with pSSBIO32 failed to generate colonies after 3 days incubation under the selective transconjugant plates. Figure 6C and D demonstrates colony formation from the conjugation assay, where *E. coli* was allowed an incubation of 12 h and 24 h, respectively, without selective conditions before subjecting them to selection. This provides evidence that *R. palustris* is at least capable of interspecies conjugation with other gram-negative bacteria. Given that *R. palustris* is capable of interspecies conjugation with mobilizable plasmids, this raises the possibility that its endogenous T4SS impacts plasmid stability through mobilization-dependent mechanisms and highlights a key mechanistic distinction between conjugation-competent hosts such as *R. palustris* and conjugation-incompetent hosts such as *E. coli*.

### MobV mutational effects on heterologous gene expression and plasmid copy number

**Fig 7:**
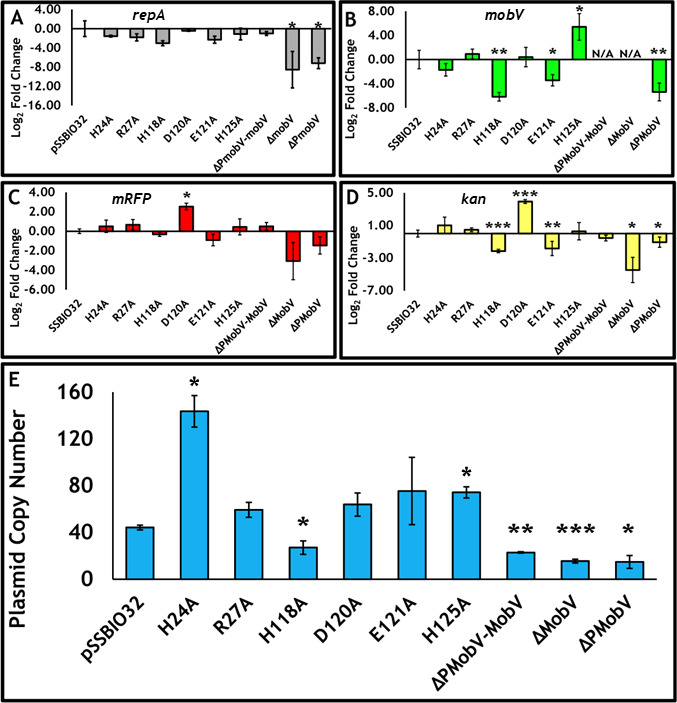
Expression rates of the various plasmid-based protein-encoding genes compared to the pSSBIO32 control containing unmodified *mobV*, including (**A**) the BBR1 replicon, *repA*, (**B**) the *mobV* mutants, (**C**) *mrfp*, and (**D**) the kanamycin selection marker. Error bars represent one standard deviation for *n* = 3, and error bars for pSSBIO32 represent standard deviation for the positive control, and a relative Log_2_ fold change of 0. (**E**) Plasmid copy numbers for the various plasmid constructs. Error bars represent one population standard deviation for *n* = 4. * = *P* < 0.05, ** *= P* < 0.005, and *** = P < 0.0005 for Welch’s *t*-test. Error bars represent one standard deviation.

Integrating heterologous genes on vectors, rather than the chromosome, typically causes a larger resource drain due to the higher number of copies. Smaller plasmids, such as those based off of mobilization plasmids, usually exist in the regime of tens to hundreds of copies per cell, and each copy of the plasmid requires more nucleotides, amino acids, ATP, and ribosomes to make the required proteins (33, 47, 48). With the potential for the MobV mutations to outright decrease the total plasmid copy number, as seen from the flow cytometry results as well as previous research on this topic (49, 50), this could also have effects on the total expression rate of the individual proteins also included on the vector. We therefore investigated the relative expression rate of each plasmid gene, including the *repA* encoded by the pBBR1 origin of replication, the kanamycin selection marker *kan*, *mRFP*, and the *mobV* mutants. We then compared expression rates to cultures with vectors containing the unmodified *mobV* sequence (Fig. 7A through D) and determined plasmid copy numbers for all cultures (Fig. 7E). Ideally, a mutant which selectively removed the oriT and kept the *mobV* promoter intact would have also been generated, though as a previous study suggested, the oriT is incorporated into the promoter sequence. This makes the effects of the mobilization protein simply being unable to recognize the oriT difficult to disentangle from the effects of removing the promoter. Therefore, only a mutant that removes the promoter outright was generated (ΔP*mobV*).

While flow cytometry results indicated that *mrfp* expression had been seriously impacted by the modifications made to MobV, interestingly, a commensurate decrease in *mrfp* transcript was not observed through qPCR. Given that the expression rate would have a more direct effect on the actual fluorescence of mRFP than plasmid retention rates, the higher correlation between the *mrfp* expression data and fluorescence intensity rather than retention rates is expected. However, no RT-qPCR result strongly correlated with the results obtained through flow cytometry, with the strongest correlation existing between bbr1 and the average fluorescence intensity of *R. palustris* cultures on day 12, exhibiting a Pearson correlation = 0.26 and *R*^2^ = 0.07. The low correlation overall is indicative that the expression rate is not the only factor in determining protein abundances in *R. palustris* for plasmid protein-encoding genes. Likewise, *bbr1* expression does not directly reflect retention rates, with *R*^2^ = 0.002 and Pearson = 0.04. The ΔP*mobV* (log_2_ fold change [*bbr1*] = −7.73 ± 3.81) and ΔP*mobV-mobV* (log_2_ fold change [*bbr1*] = −6.38 ± 1.12) mutants, however, produced a massive significant decrease in pBBR1 replicon expression, despite being upstream on the opposite strand.

The MobV protein participates in plasmid relaxation near the origin of replication, with the encoded oriT recognized by MobV being 100 bp upstream of the start of the origin of replication sequence. This could produce a twofold effect, whereby the alteration of the sequence near the origin of replication and the removal of the protein that relaxes the plasmid can severely decrease the transcription rate of the origin’s replicon. This would be consistent with the hypothesis that MobV is improving relaxation rates and overall increasing plasmid stability with *R. palustris*. The hypothesis that MobV operates using the same mechanism as MobM is not supported, however, as the inactivation of H24, H118, or H125 by changing to alanine does not produce similar significant results. The fact that retention rates from flow cytometry did not reflect this could indicate that the cultures had not yet lost the plasmid and that the plasmid retention period is longer than expected. The fact that the removal of both the promoter and the gene does not constitute a significant change in the expression of *bbr1* is unexpected, as the removal of either part constitutes a large decrease in *bbr1* transcript. This could be due to conformational or topological changes to plasmid DNA structure, which could prevent *bbr1* transcription. *mobV* transcription appears to be affected by several of the mutations as well (Fig. 7B). Given that *mobV*’s primary enzymatic function is binding to the oriT and performing single-stranded cleavage, it is possible that ablating mutation to *mobV* produces an autoregulatory effect. The autoregulatory effect could be produced as a result of the inactivated mutant *mobV* binding and failing to cleave the DNA and simply remaining docked with the plasmid DNA. While there is no conclusive evidence of this happening between MobV and pSSBIO32 when employed in *R. palustris*, the autoregulatory effect of relaxases, including the MobM protein specifically, has been precedented (51). As a result, this could explain some deleterious mutations producing an expression regulation effect both upstream and downstream, despite no mutation occurring in the promoter region.

As for the expression of *kan*, H118A (log_2_ fold change [*kan*] = −2.15 ± 0.21), E121A (log_2_ fold change [*kan*] = −1.83 ± 0.89), as well as both Δ*mobV* (log_2_ fold change [*kan*] = −4.47 ± 1.54) and ΔP*mobV* (log_2_ fold change [*kan*] = −1.06 ± 0.64), produce significant decreases in relative expression rates. Considering transcription does not necessarily stop at the stop codon of a gene, it is possible that “read-through” is occurring where transcription continues to the next protein-encoding gene. As the kanamycin selection marker is downstream of the mobilization gene, it is possible that transcription of *mobV* can cause increased transcription of *kan* as well, and that any positive or negative effects on *mobV* transcription can be propagated downstream to *kan*. This is only somewhat supported by the correlation between the two relative expression levels (*R*^2^ = 0.30 and Pearson = 0.55).

A more direct measurement of plasmid retention and stability over time is to measure plasmid copy numbers. The most prevalent method by which bacteria maintain plasmids is by possessing a high number of plasmids per cell, which would statistically guarantee that both daughter cells would retain the plasmid upon division. This method is more common with bacteria that possess smaller plasmids that can be maintained at higher copy numbers. Figure 7E displays the plasmid copy numbers obtained for *R. palustris* cultures containing plasmids with *mobV* mutants. Generally, the higher the copy number, the higher the implied stability. A statistically significant decrease in plasmid copy number would then represent lowered plasmid stability. Of the developed mutants, H118 (27.10 ± 5.74), ΔP*mobV-mobV* (22.86 ± 0.63), Δ*mobV* (15.49 ± 1.71), and ΔP*mobV* (14.81 ± 5.52) all produced significantly decreased plasmid copy numbers compared to the positive control (44.27 ± 2.00). The removal of the parts of the mobilization protein-encoding gene expectedly produced large reductions in plasmid copy numbers, but the fact that H118A reduces copy number also correlates with the results seen from *kan* and *mobv* expression rates.

The most interesting trend from the relative expression rates and plasmid copy number results is that both H24A and H125A actually appear to significantly increase the plasmid copy number rate per cell, with H24A having a very large increase to 143 ± 13.5 copies. This is highly unlikely as pBBR1-based plasmids are not usually present in the regime of upwards of 100’s of plasmids per cell (41). This would cause a large metabolic burden on the cell and, moreover, is not corroborated by the flow cytometry results, the plasmid relaxation assay, or the relaxation assay. From the covalent pull-down assays, the removal of H24 and H125 individually appears to disable MobV’s ability to relax the plasmid, but that is not reflected in the transcription rates, nor in the total plasmid copy number. The most likely explanation is that these specific mutants are interfering with the qPCR assay itself, as an inactivated enzyme attached to the DNA can be inflating the qPCR signal (49).

### Conclusions

This study investigated the plasmid retention effects of the MobV protein when employed on pBBR1 plasmids inserted into *R. palustris* through four separate means. Mutations were made to MobV based on both homology to MobM as well as several previously reported critical amino acids to determine their effect on relaxase activity, as well as reliable plasmid expression. Using flow cytometry, we elucidated plasmid retention rates and the effects of MobV on mRFP fluorescence when the various MobV mutants were employed on the plasmid. The largest differences were produced when these mutants were employed in two different gram-negative hosts. In *E. coli*, both the site-mutants and the deletion mutants had little to no effect on expression rates after 3 days of culturing, whereas in *R. palustris*, fluorescence intensity was significantly, deleteriously lowered. While retention largely remained unchanged depending on the MobV mutations, overall culture plasmid gene expression was significantly decreased.

**Fig 8:**
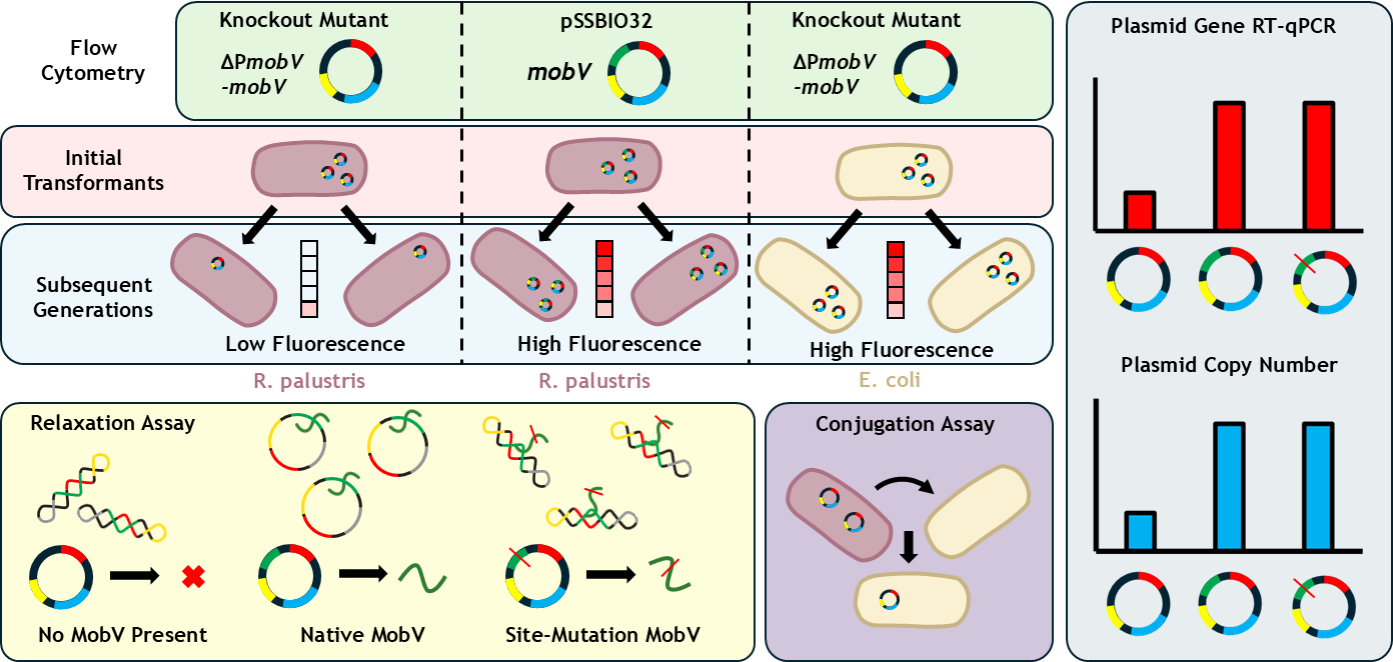
Summary of results obtained from this study. Flow cytometry was used to determine plasmid retention rates and fluorescence intensity of plasmid-based mRFP. Both relaxation and conjugation functional assays were employed to determine the mechanics of MobV performance in *R. palustris*. Finally, RT-qPCR was utilized to determine the effects of MobV protein activity on plasmid expression and copy number.

From our analyses, MobV plays a large role in maintaining high plasmid expression in *R. palustris* (Fig. 8), with effects that are not explicitly tied to plasmid retention alone, and are likely host dependent. The covalent pull-down assay revealed that MobV was indeed performing the plasmid relaxation, and the histidine knockouts did remove some relaxation activity, but the removal of the histidines did not completely ablate its activity. A conjugation assay was also then performed to determine if MobV was indeed allowing *R. palustris* to perform conjugation via its two endogenous T4SS operons. RT-qPCR results unfortunately did not produce corollary results in terms of reduced expression rates when compared to the flow cytometry results. However, the individual amino-acid mutations did reveal that MobV mutations had some, if not an inconsistent, regulatory effect on plasmid expression. The presence of MobV on the plasmid, however, did demonstrate that it is necessary for a reliable high number of plasmids overall, as each of the deletion mutants also produced a deleterious effect on copy number. The same effect was not seen with the individual active-site mutants, with some mutants, particularly H24A and H125A, contrarily raising plasmid copy number despite the active site ablation. Therefore, instead of a biological cause for this abnormality in the data we collected, we believe that an inactivated MobV protein that is still attached to the plasmid is potentially interfering with the qPCR assay.

While MobV does not retain complete homology with MobM, the 3D structures between the two proteins are nearly identical, and it is possible that the differences in the sequences may compensate for an ablation that occurs to the mobilization protein. The data indicate that MobV acts via DNA relaxation near the replication origin or plasmid origin of transfer, altering DNA topology and thereby influencing the initiation of replication or transcription. This suggests a tunable relaxase module that can be engineered to fine-tune plasmid copy number or gene expression in synthetic constructs. From our RT-qPCR data, our study finds that MobV may be influencing other plasmid genes primarily through influencing topology, and then secondarily through copy number. We conclude that MobV is necessary for both stability and plasmid expression in *R. palustris*, and mutations that inactivate MobV’s relaxation activity appear to have a large regulatory effect on the other plasmid genes.

## MATERIALS AND METHODS

### Strain construction

*Rhodopseudomonas palustris* (Molisch) van Niel (VN) BAA-98 CGA009 was obtained from the American Type Culture Collection (ATCC), and both the wild-type and any developed *R. palustris* strains were stored at −80°C in a 20% (vol/vol) glycerol solution. NEB 10-beta Competent (DH10β) *E. coli* was used for all plasmid construction, followed by transformation into Stellar Competent dam^-^/dcm^-^ *Escherichia coli* (ATCC) to remove demethylation before insertion into *R. palustris*. DH10β *E. coli* and all developed strains were stored with 15% (vol/vol) glycerol. *R. palustris* and *E. coli* strains retrieved from storage were first grown on solid 112 VN media and solid LB (Miller, AMRESCO), respectively, with appropriate antibiotics. All incubation was performed using a New Brunswick Innova 42 incubator.

All plasmids were constructed using Blunt End Ligation (52) using NEB T4 Polynucleotide Kinase and T4 DNA Ligase or Hot Fusion Method (53) and are listed in Table S2. SnapGene software was used in aid of visualizing and construction of plasmids. All oligonucleotides used for construction were purchased through Eurofins Genomics or Integrative DNA Technologies and are described in Table S3 and S4. Briefly, PCR was conducted using a Phusion Hot Start II Polymerase (Thermo Scientific) protocol and using a BIO-RAD S1000 Thermal Cycler. Upon verifying the correct size of the amplicon through gel electrophoresis with 1× tris-acetate-EDTA 1% agarose gels, the PCR product was then purified with the Monarch DNA Gel Extraction Kit (New England Biolabs Inc). DH10β *E. coli* was incubated aerobically overnight at 30°C and 275 rpm in 4 mL of LB media in 14 mL round-bottom tubes. *E. coli* cultures were then diluted to 1/40th OD_600_ in 4 mL of LB media and incubated for 1 h at 30°C and 275 rpm until mid-exponential growth phase. Cells were centrifuged at 4,000 × *g* for 15 min to remove supernatant and then washed with 4 mL of autoclaved water. Cells were washed twice before transformation. After washing, cells were transformed with assembled hot fusion or blunt-end ligation product through electroporation at 2.5 kV using a BIO-RAD Micropulser. Electroporated *E. coli* cells were then incubated in 0.450 mL of LB media at 30°C and 275 rpm for 1.5 h before streaking onto LB plates supplemented with 30 μg/mL kanamycin, and subsequent incubation at 30°C overnight. Colonies were selected from the plates and grown in 4 mL of fresh 30 μg/mL kanamycin-supplemented LB media overnight. All DH10β *E. coli* cultures were then stored at −80°C in a 15% (vol/vol) glycerol solution. Constructed plasmids from *E. coli* cultures were harvested using the Purelink Quick Plasmid Miniprep Kit (Invitrogen) and were then verified via whole plasmid sequencing by Plasmidsaurus (https://plasmidsaurus.com/).

*R. palustris* cultures were constructed using largely the same protocol, with the exception of the required incubation times before and after transformation. Liquid wild-type *R. palustris* cultures were streaked from solid media and suspended in 4 mL of VN media for 24–48 h at 30°C, before a 1/4th dilution into fresh VN media and incubated overnight. After electroporation, streaked transformations were then incubated at 30°C for up to 7 days to allow for colonies to appear, before streaking the colonies onto fresh VN plates supplemented with appropriate antibiotic. After solid cultures were grown, colonies from the second transformation plates were transferred into liquid VN cultures with supplemented antibiotic and incubated at 275 rpm and 30°C for 36 to 48 h. Transformed cultures were stored at −80°C in a 20% (vol/vol) glycerol solution. NEB BL21(DE3) *E. coli* heat-shock transformations were carried out as per their protocol.

### Flow cytometry

Both liquid *R. palustris* and *E. coli* cultures containing MobV mutants were inoculated from seed cultures to an initial OD_660_ and OD_600_ = 0.2, respectively. *R. palustris* and *E. coli* cultures were supplemented with kanamycin with final concentrations of 300 μg/mL and 30 μg/mL, respectively, and were both incubated at 275 rpm and 30°C. *R. palustris* cultures were incubated for 4, 8, and 12 days after inoculation before submission to flow cytometry, and *E. coli* cultures were incubated 1, 2, and 3 days after inoculation before submission. After incubation, cultures were then resuspended in 150 μL 0.85% NaCl at 0.01 OD_660_ and loaded into a Fisherbrand clear, polystyrene, 350 μL, flat-bottom 96-well plate. The cultures were analyzed by a Beckman Coulter CytoFLEX LX flow cytometer. mRFP was excited by a 561 nm yellow/green laser, and the emission was collected using a 610/20 nm bandpass filter. A total of 10,000 bacterial events were collected per sample. *R. palustris* cultures transformed with MobV mutants for the purposes of measuring plasmid retention and mRFP fluorescence rates were initially grown from a seed culture by inoculation into 112 Van Niel’s media to OD_660_ = 0.05 and OD_600_ = 0.25, respectively. These cell cultures were grown until stationary, then diluted to concentrations ranging from 1/100th to 1/10,000th of the initial OD to prevent clumping, into a 96-well plate, with 200 μL of 1× PBS and 0.85% NaCl (wt/vol). Negative controls consisting of wild-type cultures and positive controls consisting of the respective host culture transformed with pSSBIO32 were used to determine fluorescence thresholds, whereby cells with fluorescent intensities were assessed. For *R. palustris*, a fluorescence intensity threshold of roughly 3,500 a.u. was used to determine if an individual cell had retained a plasmid. Plasmid retention rates were then derived by taking the proportion of cells of the entire culture that had a higher fluorescence intensity than the threshold. No bovine serum albumin was added as its addition masked the fluorescence of mRFP. Flow cytometry results are available in Data S1and S2.

### Conjugation assay

Conjugation assays were used to determine if *R. palustris*’ ability as a donor strain to perform horizontal gene transfer as a means of propagating the plasmid containing *mobV*. For the conjugation assay used in this study, DH10β *E. coli* transformed with a gentamycin resistance marker-carrying plasmid was chosen as the recipient strain. DH10β *E. coli* lacks a T4SS, which prevents it from acting as a donor strain. Both strains were harvested at the exponential phase for the conjugation assay. The donor strain of *R. palustris*, transformed with pSSBIO32, which contained the kanamycin resistance marker, was streaked from solid LB/Kan plates into liquid VN/Kan media and incubated at 30°C for 48 h. The gentamycin-resistant recipient strain of DH10β *E. coli* was suspended into gentamycin-supplemented LB media at 10 μg/mL and incubated at 30°C for 12 h. Both strains were harvested, centrifuged at 4,000 × *g* for 15 min, and washed once with 1× PBS.

Three sets of solid media were prepared for the conjugation assay. VN plates supplemented with 300 μg/mL of kanamycin were used for positive controls to ensure proper growth of the donor *R. palustris* containing the pSSBIO32 plasmid. LB plates supplemented with 10 μg/mL gentamycin were likewise used to ensure healthy growth of the recipient DH10β *E. coli*. Transconjugant plates were composed of LB and were supplemented with 50 μg/mL of kanamycin as well as 5 μg/mL of gentamycin (LB/Kan/Gent). LB was selected as the media for the transconjugant plates, as *R. palustris* is also unable to grow solely on LB, presenting a media incompatibility in addition to the low amount of gentamycin selection pressure present in the transconjugant plates. The reduced antibiotic concentrations were also used as both antibiotics are aminoglycosides, resulting in potential additive translational stress with both present in the plate. As a result, only the gentamycin-resistant *E. coli* which has received the kanamycin selection marker will be able to form colonies on the transconjugant plates.

A total of 100 μL of washed pSSBIO32 *R. palustris* cells were streaked onto the kanamycin-supplemented VN plates, and growth was confirmed following 72 h of incubation at 30°C. A total of 100 μL of washed *E. coli* was likewise streaked onto the gentamycin-supplemented LB plates, and growth was confirmed following 24 h of incubation at 30°C. Conjugation between *E. coli* and *R. palustris* was performed by preparing mixtures containing 10:1, 30:1, and 100:1 recipient-to-donor ratio dilutions. These dilutions were prepared as *E. coli* possesses a much reduced doubling time compared to that of *R. palustris*. A total of 400 μL of each dilution was prepared with an OD_660_ = 1.0 of *R. palustris* as a basis and the appropriate OD_600_ of *E. coli* for each dilution. For recovery, non-selective VN plates were prepared by segmenting the plates into six equal sections. A total of 50 μL of each dilution was then spotted onto the plates on each section, allowing the mixtures to dry before incubation at 30°C for 12 h and another set of plates for 24 h. Negative controls were also prepared by spotting solely the washed pSSBIO32 *R. palustris* cells and solely the *E. coli* cells.

Transconjugant plates were segmented into three equal sections before streaking spots from the non-selective plates. After 12 and 24 h incubations, respectively, for the transconjugant plates, the spots were resuspended and streaked onto the transconjugant plates. The negative controls were also streaked onto transconjugant plates to ensure selective conditions and no colony formation. After streaking, transconjugant plates were incubated for a further 72 h before observing colony formation.

### Protein expression, purification, and covalent pull-down assays

pET-24b vectors containing T7 promoters and kanamycin resistance markers were purchased from AddGene and used for overexpression of MobV and MobV histidine mutants. pET-24b vectors containing MobV mutants were constructed using the aforementioned protocol and were subsequently inserted into BL21(DE3) *E. coli*. Each MobV construct was ensured to exist downstream of the T7 promoter, and a 6xHistag was placed at the 3′ end of each MobV mutant as previously described to ensure the tag does not interfere with the plasmid binding mechanism. A total of 4 mL of liquid LB/kan^30^ cultures of constructed BL21 strains was inoculated from frozen stock and grown overnight at 30°C at 275 rpm. BL21 cultures were then diluted to 1/50th in 4 mL of LB/kan^30^ media and grown for 4 h at 30°C and 275 rpm to an exponential growth phase. A total of 2 mL of this culture was removed, and 2 mL of fresh LB/kan^30^ with a final IPTG concentration of 1 mM was added. The cultures were incubated at room temperature overnight at 275 rpm before harvesting. Protein purification was performed with a Qiagen Ni-NTA spin kit, and extracts were visualized using SDS-PAGE to verify the extracted protein size. Extracts were then dialyzed using a Thermo Scientific Slide-A-Lyzer 10 MWCO 15 mL conical tubes, with a storage buffer composition of 5% glycerol, 300 mM NaCl, 1 mM EDTA, and 50 mM Tris-HCl at pH 7.6. Protein extracts were then stored at −20°C for up to 2 months. Protein quantitation was performed using a Pierce Dilution-Free Rapid Gold BCA Protein Assay.

The covalent pull-down assay was performed as described previously (12). Briefly, supercoiled plasmid was purified from 1L of DH10β *E. coli* through alkaline lysis. A total of 500 ng of supercoiled plasmid was then added to 20 μL reactions (25 mM Tris–HCl pH 7.6, 0.1 mM EDTA, 10% glycerol [vol/vol], 1 mM dithiothreitol, NaCl 50 mM, and 8 mM MnCl_2_) containing 500 nM, 250 nM, and 100 nM of purified MobV proteins. Reactions were then incubated at 30°C for 20 min and were analyzed on a 1% agarose gel with 10,000× SYBR Safe DNA Gel Stain.

### RNA extraction

RNA extractions were performed as previously described (34, 42). Briefly, the desired *R. palustris* cultures were grown to a mid-exponential stage before immediately harvesting and starting RNA extraction through the TRIzol method. RNAlater or other RNA stabilizing agents were not used, as RNAlater appeared to prevent RNA pellet precipitation with isopropanol, and mRNA from all cultures was instead extracted when harvested. mRNA extracts were stored at −80°C. DNA from RNA extracts was removed using the Invitrogen TURBO DNase kit. RNA extracts were then converted to cDNA by reverse transcription using the Multiscribe Reverse Transcriptase protocol.

### RT-qPCR and plasmid copy number quantification

RT-qPCR oligonucleotides were purchased through Eurofins Genomics or IDT and are described in Table S3. Briefly, primers were designed to have a length between 70 and 200 bp depending on the GC content of the amplicon of each gene of interest, with shortening length corresponding with increasing GC content. Primer sets were initially evaluated through 20 μL PCR reactions using GoTaq polymerase, with primer concentrations of 50–500 nM and using ~100 ng of genomic DNA as a substrate. Negative controls for each concentration were prepared by the addition of water instead of genomic DNA. After PCR, reactions were visualized on 2% agarose gels. Primer concentrations that produced a band for the positive controls and no band for the negative controls were then considered for primer efficiency testing.

Primer concentrations that met these criteria were used to inform primer efficiency tests. Primer efficiency was determined by preparing qPCR reactions with PowerUp SYBR Green Master Mix (Life Technologies), with a 5× dilution series of cDNA (500–0.0061 ng/μL). qPCR reactions were run in an Eppendorf Mastercycler Realplex. Thermocycler settings for qPCR reactions were 50°C for 2 min, 95°C for 2 min, 40 cycles of 95°C for 15 s, and 60°C for 1 min, with a melting curve step (60°C for 1 min, rising at a rate of 1.5°C for 20 min, staying at 95°C for 15 s) to ensure a single amplicon. Non-template controls (NTC) in triplicate were included for each reaction to ensure a lack of unintended PCR products for each target gene.

The Eppendorf Mastercycler Realplex automatic baseline calculator was used to calculate the baseline for samples. Cycle threshold (CT) values were then obtained from the Eppendorf Mastercycler Realplex. Primer efficiency for each primer set reaction was determined by plotting the log_10_ of cDNA copies vs the CT value for each set of reactions. Linear regression was used to ensure linearity of the plot. The slope of the graph determined the primer efficiency of the reaction. The Thermo Fisher Scientific qPCR calculator was used to determine primer efficiency for each reaction set. Using this method, all primers were ensured to have a primer efficiency between 90% and 110% and are reported in Table S3. qPCR reactions for two biological replicates and two technical replicates were then run in the Eppendorf Mastercycler Realplex using the same settings as for primer efficiency determination. Primer efficiency results are outlined in Data S4. All qPCR reactions were performed using PowerUp SYBR Green Master Mix (Life Technologies). NTC controls in triplicate were also run. The relative mRNA concentration was calculated per the following equation, GOI is the gene of interest, and HK is the housekeeping gene.


$$Log_{2}\;Fold\;Change=\left(2^{-\left(CT^{GOI}-CT^{HK}\right)}\right)avg,$$

Where

CT^GOI^ = CT of gene of interest

CT^HK^ = CT of housekeeping gene

Ct values for each of the plasmid-based genes are available in Data S5.

### Plasmid copy number

qPCR with gDNA was used to determine the absolute plasmid copy number ratio of both native and non-native plasmids relative to the chromosome as previously described (52). gDNA was extracted from *R. palustris* cultures at mid-exponential phase in duplicate using the Monarch Genomic DNA Purification Kit (New England Biolabs Inc.). This kit uses alkaline lysis and a silica membrane column that have been shown to efficiently extract and purify both plasmids and chromosomes (53, 54). Amplified PCR product using primers was diluted to 10^9^ copies of amplicon/μL per the following equation.


$$Amplicon\;copy\;number=\;\frac{\left(6.02*10^{23}\frac{copies}{mol}\right)\left(gDNA\frac{ng}{\mu L}\right)}{\left(amplicon\;length\left(bp\right)\right)*\left(660\frac{g}{mol*bp}\right)}.$$

An external standard curve was constructed using a 5× dilution series of this diluted PCR product, starting with 10^7^ copies/μL in triplicate. The primer efficiency was determined as outlined in the previous section. qPCR reactions for two (2) biological replicates and two (2) technical replicates were then run in the Eppendorf Mastercycler Realplex using the same settings as for primer efficiency determination. Three (3) non-template controls in triplicate were also run. The standard curve was used to determine the number of copies of each amplicon. The plasmid copy number relative to the chromosome was then determined, and Ct values are available in Data S3.

### Statistical methods

All statistical analyses (Welch’s *t*-test) were performed using Microsoft Excel. A *P* value < 0.05 was considered to be significant. Flow cytometry was performed in biological triplicate, RT-qPCR of individual plasmid-based genes was performed with *n* = 4 and technical duplicate, and plasmid copy number was performed in biological triplicate and technical duplicate. All error bars represent the population standard deviation and were calculated using Excel.

## Data Availability

The complete plasmid sequence for pSSBIO32 has been deposited in GenBank under accession number PZ463017.

## AFTER EPUB

*[This article was published on 16 July 2026 with an incorrect grant number in Funding. The error was corrected in the current version, posted on 23 July 2026.]*

## SUPPLEMENTAL MATERIAL

Text S1 spectrum.03819-25-s0001.fasta pSSBIO32 sequence.

Data S1 spectrum.03819-25-s0002.xlsx Raw data for *R. palustris* flow cytometry results.

Data S2 spectrum.03819-25-s0003.xlsx Raw data for *E. coli* flow cytometry results.

Data S3 spectrum.03819-25-s0004.xlsx

Data S4 spectrum.03819-25-s0005.xlsx Primer efficiency results.

Data S5 spectrum.03819-25-s0006.xlsx RT-qPCR results obtained for various expression rates for plasmid-based genes.

Supplemental legends spectrum.03819-25-s0007.docx Legends for Data S1 to S5.

Supplemental tables spectrum.03819-25-s0008.docx Tables S1 to S4.

ASM does not own the copyrights to Supplemental Material that may be linked to, or accessed through, an article. The authors have granted ASM a non-exclusive, world-wide license to publish the Supplemental Material files. Please contact the corresponding author directly for reuse.
