# Supplementary material for: Performance of the pBHR1 mobilization protein MobV and its role in stable plasmid expression in Rhodopseudomonas palustris CGA009
Source: Microbiol Spectr. 2026 Jul 16;14(8):e03819-25. doi: 10.1128/spectrum.03819-25 (PMC13436267; doi:10.1128/spectrum.03819-25)
Supplement: Supplementary file 1 [file spectrum.03819-25-s0007.docx]

Description of additional supplementary files

Title: Supplementary Document 1.

Description: Statistics for flow cytometry results, plasmids used in this work, primers for plasmid construction used in this work, RT-qPCR primers used in this work

Title: Supplementary Data 1

Description: Raw data for *R. palustris* flow cytometry results.

Title: Supplementary Data 2

Description: Raw data for *E. coli* flow cytometry results.

Title: Supplementary Data 3

Description: Ct values and plasmid copy number calculations

Title: Supplementary Data 4

Description: Primer efficiency results

Title: Supplementary Data 5.

Description: RT-qPCR results obtained for various expression rates for plasmid-based genes
