# Supplementary material for: Performance of the pBHR1 mobilization protein MobV and its role in stable plasmid expression in Rhodopseudomonas palustris CGA009
Source: Microbiol Spectr. 2026 Jul 16;14(8):e03819-25. doi: 10.1128/spectrum.03819-25 (PMC13436267; doi:10.1128/spectrum.03819-25)
Supplement: Supplementary file 2 [file spectrum.03819-25-s0008.docx]

**Supplementary Information for**

**Performance of the pBHR1 Mobilization Protein MobV and Its Role in Stable Plasmid Expression in Rhodopseudomonas palustris CGA009**

Department of Chemical and Biomolecular Engineering, University of Nebraska-Lincoln, NE 68588, United States

^*^Corresponding Author: [rsaha2@nebraska.edu](mailto:rsaha2@nebraska.edu)

**Table 1:** Plasmid retention rates and average fluorescence intensities of *E. coli* & *R. palustris* transformed with pSSBIO32, and pSSBIO32 derivates containing mutations to MobV. Significance represents differences between mutant strains and the pSSBIO32 positive control. * = *P* < 0.05, ** = *P* < 0.005, *** = *P* < 0.0005 for n = 3. Data can be found in Supplementary Data 1 & 2.

|  | ***E. coli*** | | | | | | | | | | | |
| --- | --- | --- | --- | --- | --- | --- | --- | --- | --- | --- | --- | --- |
|  | **Plasmid Retention** | | | | | | **Fluorescence Intensity** | | | | | |
|  | **Day 1** | | **Day 2** | | **Day 3** | | **Day 1** | | **Day 2** | | **Day 3** | |
| **Strain** | **Average** | **Significance** | **Average** | **Significance** | **Average** | **Significance** | **Average (10^3^ a.u.)** | **Significance** | **Average (10^3^ a.u.)** | **Significance** | **Average (10^3^ a.u.)** | **Significance** |
| pSSBIO32 | 0.944±0.015 | NA | 0.867±0.007 | NA | 0.886±0.005 | NA | 43.2±7.9 | NA | 43.7±1.8 | NA | 50.0±3.1 | NA |
| H24A | 0.738±0.051 | * | 0.826±0.020 | - | 0.805±0.027 | * | 8.6±2.7 | * | 13.3±4.1 | ** | 13.6±5.7 | ** |
| R27A | 0.526±0.092 | * | 0.783±0.069 | - | 0.710±0.073 | - | 4.1±1.5 | * | 8.8±2.3 | *** | 8.4±2.4 | *** |
| H118A | 0.776±0.053 | * | 0.828±0.015 | * | 0.803±0.025 | * | 7.8±0.8 | * | 15.1±1.2 | *** | 15.1±1.9 | *** |
| D120A | 0.690±0.099 | - | 0.817±0.009 | * | 0.794±0.015 | * | 7.3±1.8 | * | 14.1±1.0 | *** | 14.3±1.7 | *** |
| E121A | 0.652±0.040 | * | 0.818±0.008 | ** | 0.798±0.006 | *** | 6.3±2.3 | * | 13.9±0.7 | ** | 14.1±0.7 | ** |
| H125A | 0.732±0.101 | - | 0.727±0.065 | - | 0.686±0.113 | - | 10.0±3.7 | * | 13.8±1.0 | *** | 11.8±3.8 | ** |
| ΔP*mobV -mobV* | 0.837±0.096 | - | 0.829±0.011 | * | 0.829±0.003 | *** | 23.9±11.9 | - | 25.0±11.5 | - | 16.9±0.3 | ** |
| Δ*mobV* | 0.679±0.181 | - | 0.820±0.037 | - | 0.723±0.062 | - | 16.3±17.2 | - | 21.8±18.3 | - | 7.6±0.9 | ** |
| ΔP*mobV* | 0.690±0.072 | * | 0.776±0.042 | - | 0.788±0.017 | * | 9.3±4.4 | * | 13.6±4.2 | ** | 19.7±1.0 | ** |
|  | ***R. palustris*** | | | | | | | | | | | |
|  | **Plasmid Retention** | | | | | | **Fluorescence Intensity** | | | | | |
|  | **Day 1** | | **Day 2** | | **Day 3** | | **Day 1** | | **Day 2** | | **Day 3** | |
| **Strain** | **Average** | **Significance** | **Average** | **Significance** | **Average** | **Significance** | **Average (10^3^ a.u.)** | **Significance** | **Average (10^3^ a.u.)** | **Significance** | **Average (10^3^ a.u.)** | **Significance** |
| pSSBIO32 | 0.994±0.002 | NA | 0.995±0.002 | NA | 0.993±0.006 | NA | 267±17 | NA | 159±5 | NA | 133±3 | NA |
| H24A | 0.993±0.001 | - | 0.996±0.002 | - | 0.998±0.002 | - | 134±37 | * | 91±8 | ** | 107±36 | - |
| R27A | 0.991±0.003 | - | 0.995±0.001 | - | 0.998±0.001 | - | 165±18 | ** | 168±20 | - | 113±22 | - |
| H118A | 0.996±0.003 | - | 0.996±0.001 | - | 0.997±0.001 | - | 129±23 | ** | 145±17 | - | 121±30 | - |
| D120A | 0.991±0.002 | - | 0.996±0.001 | - | 0.997±0.002 | - | 121±34 | * | 124±36 | - | 124±4 | - |
| E121A | 0.994±0.001 | - | 0.996±0.001 | - | 0.997±0.001 | - | 153±18 | ** | 135±31 | - | 136±22 | - |
| H125A | 0.993±0.001 | - | 0.995±0.000 | -- | 0.994±0.003 | - | 157±18 | ** | 149±24 | - | 147±24 | - |
| ΔP*mobV -mobV* | 0.994±0.004 | - | 0.996±0.003 | - | 0.993±0.005 | - | 129±31 | * | 111±38 | - | 97±9 | * |
| Δ*mobV* | 0.995±0.002 | - | 0.994±0.001 | - | 0.980±0.026 | - | 139±13 | ** | 128±10 | * | 91±13 | * |
| ΔP*mobV* | 0.993±0.003 | - | 0.995±0.001 | - | 0.992±0.008 | - | 123±15 | ** | 127±38 | - | 112±26 | - |

**Supplementary Table 2**: Plasmids used in this work.

| **Name** | **Parts** | **Type and Source** |
| --- | --- | --- |
| BBR1-kan-mRFP-MobV (Control Vector, pSSBIO32) | pBBR1 replicon; *kan^R^; mrfp; mobv* | Replicative  This Study |
| BBR1-kan-mRFP-ΔPMobV-MobV | pBBR1 replicon; *kan^R^; mrfp;* | Replicative  This Study |
| BBR1-kan-mRFP-ΔPMobV | pBBR1 replicon; *kan^R^; mrfp; mobv(ΔPmobv)* | Replicative  This Study |
| BBR1-kan-mRFP-ΔMobV | pBBR1 replicon; *kan^R^; mrfp; mobv(Δmobv)** | Replicative  This Study |
| BBR1-kan-mRFP-MobV(H24A) | pBBR1 replicon; *kan^R^; mrfp; mobv*(H24A) | Replicative  This Study |
| BBR1-kan-mRFP-MobV(R27A) | pBBR1 replicon; *kan^R^; mrfp; mobv*(H24A) | Replicative  This Study |
| BBR1-kan-mRFP-MobV(H118A) | pBBR1 replicon; *kan^R^; mrfp; mobv*(H24A) | Replicative  This Study |
| BBR1-kan-mRFP-MobV(D120A) | pBBR1 replicon; *kan^R^; mrfp; mobv*(D120A) | Replicative  This Study |
| BBR1-kan-mRFP-MobV(E121A) | pBBR1 replicon; *kan^R^; mrfp; mobv*(E121A) | Replicative  This Study |
| BBR1-kan-mRFP-MobV(H125A) | pBBR1 replicon; *kan^R^; mrfp; mobv*(H125A) | Replicative  This Study |

*Only the gene is removed for this construct, *mobv*’s natural promoter is left intact.

**Supplementary Table 3**: RT-qPCR primers used in this work

| **Primer Pairs** | **Sequence** | **Primer Conc.** | **cDNA Conc.** | **Primer Efficiency (%)** | **R^2^** |
| --- | --- | --- | --- | --- | --- |
| 16S rRNA F | GCTTAACACAT  GCAAGTCGAAC | 40 | 0.2 | 100.7 | 0.976 |
| 16S rRNA R | TCATCCTCTCA  GACCAGCTAC |  |  |  |  |
| Kan^R^ F | CAGTCATAGCCG  AATAGCCTCTC | 100 | 25 | 97.64 | 0.979 |
| Kan^R^ R | CAAAGTAAACTGG  ATGGCTTTCTTGC |  |  |  |  |
| dxs F | CTTCTCGACA  CCATTCGCAC | 100 | 25 | 101.3 | 0.999 |
| dxs R | ACATAGTGCAG  TGCGGTAGTC |  |  |  |  |
| mRFP F | CGTTTCAAAGTTC  GTATGGAAGGTTC | 100 | 25 | 104.9 | 0.996 |
| mRFP R | GTGTTTAACGTAAG  CTTTGGAACCGTAC |  |  |  |  |
| qRepA F | CCAGAGAAATC  GGCATTCAAGC | 100 | 25 | 99.84 | 0.993 |
| qRepA R | ATGAGCTTGGAA  AGTGTCTTCTGG |  |  |  |  |
| Mobv F | GCGATCATGC  GATGCAAGAAG | 100 | 25 | 105.6 | 0.985 |
| Mobv R | GCTTCTCTGG  CAGCAACTCG |  |  |  |  |

**Supplementary Table 4**: RT-qPCR primers used in this work

| **Primer Pairs** | **Sequence** | | |  |  | |  |
| --- | --- | --- | --- | --- | --- | --- | --- |
| E121 KO r | | GCCGAAACCAGCCCGCACATGACC | Mob CO F W/O Pm | | | CAGCCCGTGGATATGTGGACGATG | |
| E121 KO r | | GTCACGGTGGATGCTGGCCGTC | Mob CO F W Pm | | | GCCCCTCCCTTTTGGTGTCCAAC | |
| H118 KO r | | CGTGACGAAACCAGCCCGCACATGAC | Mob CO R | | | CACCTGGGATGAATGTCAGCTACTGG | |
| H118 KO f | | GGCGATGCTGGCCGTCACGATGCGATC |  |  |  |  |  |
| R27 KO r | | GCGGAGCGCGAGACGCCCAACGCTG |  |  |  |  |  |
| R27 KO f | | GTAGGCGTGCTTGAGACTGGCCGCCAC |  |  |  |  |  |
| D120 KO r | | GCCGAAACCAGCCCGCACATGACC |  |  |  |  |  |
| D120 KO f | | ACGGTGGATGCTGGCCGTCAC |  |  |  |  |  |
| H24 KO r | | GCCTACCGCGAGCGCGAGAC |  |  |  |  |  |
| H24 KO f | | GGCCTTGAGACTGGCCGCCACGTTG |  |  |  |  |  |
| H125 KO r | | GCGATGACCGCGTTCGTGGTGC |  |  |  |  |  |
| H125 KO f | | CGGGCTGGTTTCGTCACGGTG |  |  |  |  |  |
